# Peptidylarginine Deiminase of *Porphyromonas gingivalis* Modulates the Interactions between *Candida albicans* Biofilm and Human Plasminogen and High-Molecular-Mass Kininogen

**DOI:** 10.3390/ijms21072495

**Published:** 2020-04-03

**Authors:** Justyna Karkowska-Kuleta, Magdalena Surowiec, Mariusz Gogol, Joanna Koziel, Barbara Potempa, Jan Potempa, Andrzej Kozik, Maria Rapala-Kozik

**Affiliations:** 1 Department of Comparative Biochemistry and Bioanalytics, Faculty of Biochemistry, Biophysics and Biotechnology, Jagiellonian University in Krakow, Gronostajowa 7, 30-387 Krakow, Poland; 2 Department of Microbiology, Faculty of Biochemistry, Biophysics and Biotechnology, Jagiellonian University in Krakow, Gronostajowa 7, 30-387 Krakow, Poland; 3 Department of Oral Immunology and Infectious Diseases, University of Louisville School of Dentistry, 501 S. Preston St, Louisville, KY 40202, USA; 4 Department of Analytical Biochemistry, Faculty of Biochemistry, Biophysics and Biotechnology, Jagiellonian University in Krakow, Gronostajowa 7, 30-387 Krakow, Poland

**Keywords:** biofilm, plasminogen, kininogen, kinins, candidiasis, periodontitis, citrullination

## Abstract

Microorganisms that create mixed-species biofilms in the human oral cavity include, among others, the opportunistic fungus *Candida albicans* and the key bacterial pathogen in periodontitis, *Porphyromonas gingivalis*. Both species use arsenals of virulence factors to invade the host organism and evade its immune system including peptidylarginine deiminase that citrullinates microbial and host proteins, altering their function. We assessed the effects of this modification on the interactions between the *C. albicans* cell surface and human plasminogen and kininogen, key components of plasma proteolytic cascades related to the maintenance of hemostasis and innate immunity. Mass spectrometry was used to identify protein citrullination, and microplate tests to quantify the binding of modified plasminogen and kininogen to *C. albicans* cells. Competitive radioreceptor assays tested the affinity of citrullinated kinins to their specific cellular receptors. The citrullination of surface-exposed fungal proteins reduced the level of unmodified plasminogen binding but did not affect unmodified kininogen binding. However, the modification of human proteins did not disrupt their adsorption to the unmodified fungal cells. In contrast, the citrullination of kinins exerted a significant impact on their interactions with cellular receptors reducing their affinity and thus affecting the role of kinin peptides in the development of inflammation.

## 1. Introduction

Microorganisms, even if they belong to different kingdoms, usually reside in the same environmental niche in the form of complex, multi-species communities, i.e., biofilms. One of the locations where biofilms can play a particularly adverse role in contributing to human health disorders is the initial part of the human digestive system which is inhabited by several thousand different microbial species [1,2]. These species exist together, creating a network of interconnections based both on mutual cooperation and protection, but also on the competition for access to nutrients and spaces to colonize [3,4]. Some of the species inhabiting the oral cavity are beneficial to the host as they are involved in maintaining overall homeostasis in this challenging environment, whilst some are opportunists or pathogens that can contribute to serious diseases [5,6]. One of these organisms, the anaerobic bacterium *Porphyromonas gingivalis*, is the key pathogen in periodontal disease which is a disorder responsible not only for gum damage and subsequent loss of teeth due to prolonged inflammation and tissue destruction but is also associated with the development of serious systemic diseases such as rheumatoid arthritis, diabetes, some cardiovascular disorders and neurodegenerative diseases such as Alzheimer’s disease [7,8,9,10,11,12,13,14]. *P. gingivalis* is present in the dental plaque in a complex assembly together with other aerobic and anaerobic bacteria, but recent studies have also shown an important role of an opportunistic fungus, *Candida albicans*, in the development of biofilms formed with the contribution of *P. gingivalis* [15,16,17,18]. In such biofilms, *C. albicans* can play a protective role for anaerobic bacteria, as the biofilm that it creates provides an anoxic environment at the site of infection, thus generating favourable conditions for the growth of *P. gingivalis* [16]. Under these conditions, both microorganisms might use arsenals of various virulence factors so that they can coexist in one ecological niche and invade host tissues as pathogens, thus ensuring their survival in the face of the host immune system response and thus further effective dissemination within the host organism [17,18]. The most important virulence factors of *P. gingivalis* include adhesive molecules―fimbriae and hemagglutinin-like proteins―hemin-binding proteins, and secreted enzymes such as cysteine proteases (gingipains) and peptidylarginine deiminase (PPAD) [19,20]. The latter enzyme modifies the C-terminal arginine residues to citrullines in peptides and proteins, so that its activity is closely related to the proteolytic action of R-gingipain, which preferentially hydrolyses peptide bonds in proteins with a preference for Arg at the P1 position and generates fragments susceptible to further modification by PPAD [21,22,23,24]. Citrullination by PPAD changes the positive charge of the modified residue to neutral, thus significantly altering the protein conformation and function. This phenomenon has been already shown to be relevant for weakening human innate immunity by disrupting the process of phagocytosis, avoiding entrapment in neutrophil extracellular traps (NETs), and impairing the action of the antimicrobial peptide LP9 [25].

The pathogenic potential of *C. albicans* is mainly dependent on the production of secreted aspartyl proteinases (SAPs), as well as numerous adhesive proteins present at the surface of the fungal cells that include typical adhesins, e.g., the family of agglutinin-like sequence proteins, and some atypical surface-exposed proteins (often classified to the “moonlighting proteins”, i.e., proteins with many different functions, often performing them at a distinct cellular location than the original one) involved in interactions with proteins of other co-inhabiting microorganisms or with host proteins [17,26,27,28]. It is well known that both of these microbial pathogens are capable of interfering with the important plasma proteolytic cascades, i.e., coagulation, fibrinolysis and the contact system, responsible for maintaining homeostasis and hemostasis in humans [29,30,31,32,33]. Thus, microbial secretory proteases can affect the activity of the enzymes in these cascades, and bacterial and fungal surface proteins can attract individual proteinaceous components of these systems to the pathogen cell surfaces for the local increase in protein concentrations and the modulation of their activity. One of such essential plasma proteins that can be seized by pathogens is plasminogen (HPG), which after its activation to plasmin is involved in the lysis of fibrin clots, regulation of the complement cascade, and activation of the matrix-metalloproteinases responsible for tissue remodelling [34,35]. Another protein, whose role in inflammation is critical and which can be used by the pathogens during infection, is high-molecular-mass kininogen (HK), which apart from its function in the intrinsic coagulation pathway is also the precursor of the vasoactive, proinflammatory peptide mediators, the kinins [36]. HPG has been shown to be effectively activated to plasmin by the *P. gingivalis* gingipains, thereby contributing to gingival tissue destruction [37]. Similarly, the adsorption of HPG at the cell surface of *C. albicans* cells greatly facilitates the dissemination of these fungal cells in the host organism, enabling them to cross the blood–brain barrier [38].

**Figure 1:**
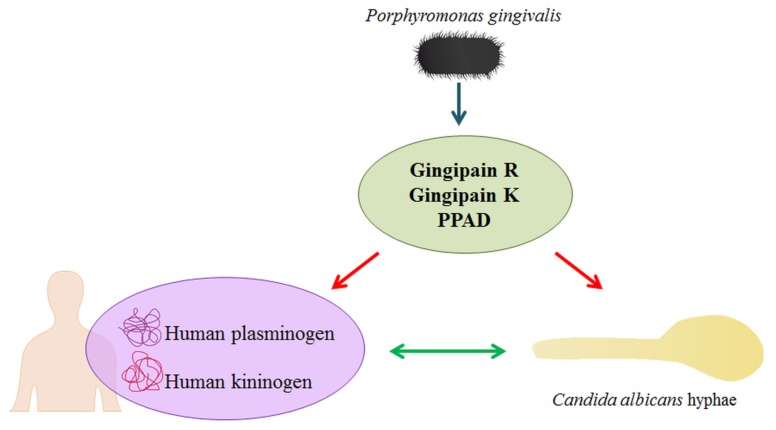
The impact of *P. gingivalis* secretory proteins on *C. albicans* and human plasma proteins. The activity of bacterial cysteine proteases (gingipains) and PPAD results in the citrullination of different proteins. During infection, in the inflammatory milieu, PPAD can citrullinate both human plasma proteins and fungal surface-exposed proteins, thereby affecting the interactions between the fungus and human proteins and modifying the level plasma protein binding by *C. albicans*.

The role of contact system assembly at the microbial cell surface for the development of inflammation should not be underestimated in terms of increasing the local concentration of the kinin precursor within the microorganism community and enhancing the possibility of producing kinins by pathogen proteinases, thus inducing the influx of plasma and nutrients to the site of infection [39,40]. As the entire repertoire of bacterial and fungal proteins, including adhesins and proteinases, is involved in this complex process of interactions between host and commensals or pathogens, the important question arises as to whether another very important virulence factor of *P. gingivalis,* PPAD, can contribute to the modulation of the complicated networks of commensal/pathogen-host relationships by modifying the bacterial and fungal proteins in the biofilm, as well as the host human proteins (Figure 1). Biofilms bring many benefits to the microorganisms that inhabit them, such as the possible joint use of an available arsenal of various virulence factors. However, it is conceivable that in such conditions, some pathogens may also shift the balance in their favour.

## 2. Results

### 2.1. Effects of Citrullination of Fungal Surface-Exposed Proteins on the Binding of Human Plasma Proteins to C. albicans Hyphae

The presence of particular *P. gingivalis* proteins secreted into the medium during culturing under anaerobic conditions was confirmed using liquid chromatography-coupled tandem mass spectrometry (LC-MS/MS). Depending on the *P. gingivalis* strain, proteases (HRgpA (gingipain R) and Kgp (gingipain K)), outer membrane proteins, and the FimA subunit of major fimbriae or hemagglutinin A were detected. Under the culture conditions used, the secretion of the PPAD enzyme was found for both wild strains W83 and American Type Culture Collection (ATCC) 33277 and a lack of this enzyme was noticed in the supernatant in the case of the investigated mutant strains *Δppad* (W83) and *Δppad* (ATCC 33277), as described previously also [22]. All of the identified extracellular bacterial proteins are listed in Table S1 in Supplementary File 1.

The citrullination of surface-exposed *C. albicans* proteins was next confirmed by cell surface shaving with trypsin and subsequent LC-MS/MS analysis after the incubation of biofilm-forming fungal hyphae with bacterial enzymes secreted into the supernatants. The confirmed presence of citrulline residues instead of arginine was based on an increase in the calculated peptide mass by 1 Da compared to the mass of the peptides derived from proteins localized at the surface of cells that had not been in contact with PPAD. To confirm that the particular peptide has been correctly identified as citrullinated and not deamidated, as the observed change in mass can be indicated for both these modifications, the sequence-specific MS/MS fragment ions were manually reviewed. Considering that both PPAD and HRgpA―capable of generating fragments terminated with an arginine residue for further modification by PPAD―were present in the preparations, obtained after cultivating *P. gingivalis* wild strains, several citrullinating sites were identified within the fungal proteins.

In total, 10 *C. albicans* surface-localized proteins were identified as modified by PPAD, including cell wall mannoprotein Mp65, endo-1,3(4)-beta-glucanase Eng1, moonlighting proteins such as enolase (Eno1) and glyceraldehyde-3-phosphate dehydrogenase (Tdh3), and the heat shock proteins Ssa1 and Ssa2. The full list of citrullinated fungal proteins identified by LC-MS/MS in this experiment, including mass spectrometric identification criteria, is provided in Table S2 in Supplementary File 2. In a previous study [16] in which the citrullination of *C. albicans* proteins exposed at the surface of hyphae that formed a mixed biofilm with *P. gingivalis* cells of wild strain W83 was tested, the modification of three proteins from this set, i.e., Eng1, Eno1 and Mp65, was also described. 

**Figure 2:**
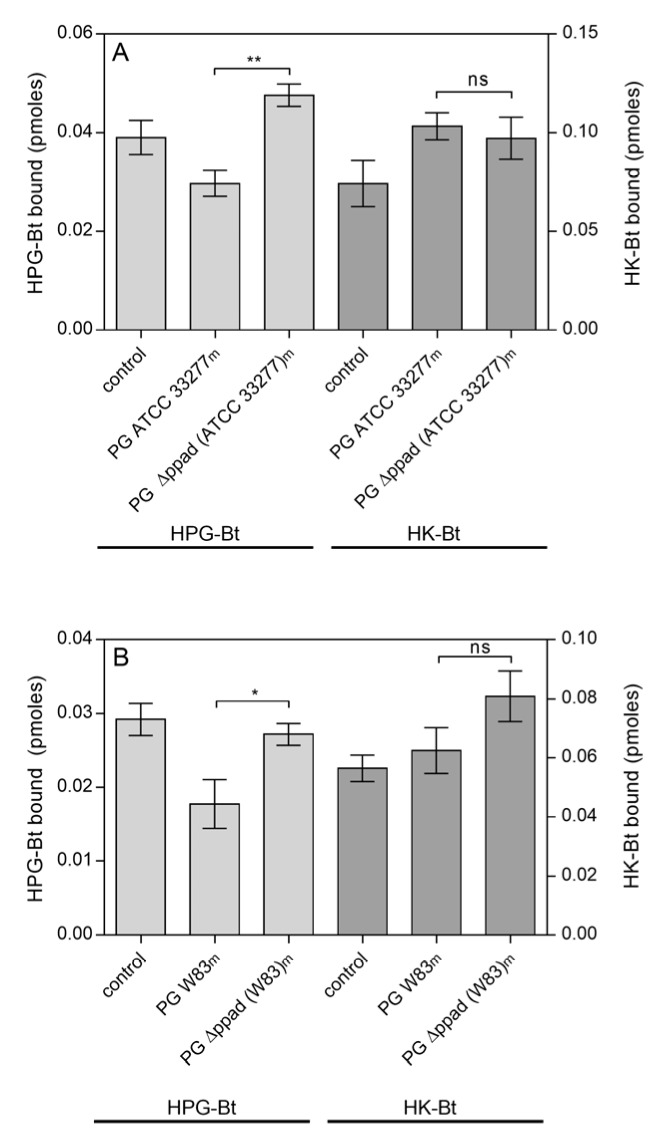
Binding of the human plasma proteins HPG-Bt and HK-Bt to *C. albicans* hyphae pretreated with enzymes secreted into the growth medium (m) by *P. gingivalis* (PG) strains: (**A**) ATCC 33277 and *Δppad* (ATCC 33277) and (**B**) W83 and *Δppad* (W83). *C. albicans* cells were incubated for 6 h in RPMI 1640 medium with the further addition of media collected after *P. gingivalis* culture. Fungal cells grown in RPMI 1640 medium with BHI or TSBY broth instead of culture supernatants from appropriate *P. gingivalis* strains served as a control. The bound biotinylated human protein was quantified using the SA-HRP/TMB detection system. Results from representative experiments are shown; bars represent the mean values ± SEM. Statistical analysis was performed by one-way ANOVA with a Tukey’s multiple comparisons test (*p* < 0.05) using GraphPad Prism software (GraphPad, LaJolla, CA, USA). Statistical significance levels are indicated: * *p*< 0.05; ** *p* < 0.01; ns, non-significant.

In our present analysis, a complete novelty in relation to the previous work is the study of the role of PPAD modification of fungal proteins in the interactions with host proteins. Next, we analysed the binding of two biotinylated human plasma proteins, plasminogen (HPG-Bt) and kininogen (HK-Bt) to the surface of fungal cells, pre-modified by enzymes secreted by bacteria (Figure 2). It should be noted however that the surface of *C. albicans* hyphae are most likely altered by the activity of the bacterial proteins found in the collected supernatants, and thus not only by PPAD but also gingipains and other proteases and enzymes (Table S1). 

A reduced level of HPG binding by *C. albicans* cells was observed after their incubation in culture media from both *P. gingivalis* wild strains ATCC 33277 (Figure 2A) and W83 (Figure 2B). If there was no PPAD activity, as in the case of *ppad* deletion mutants, no difference in the level of HPG binding was observed compared to the control, i.e., fungi grown in BHI (brain heart infusion) or TSBY (trypticase soy broth with yeast extract) medium devoid of any bacterial proteins. For HK, there were no significant differences between the levels of binding to the surface of hyphae grown in culture supernatants from *P. gingivalis* wild and mutant strains, regardless of whether it was the ATCC 33277 or W83 strain (Figure 2A,B, respectively). 

**Figure 3:**
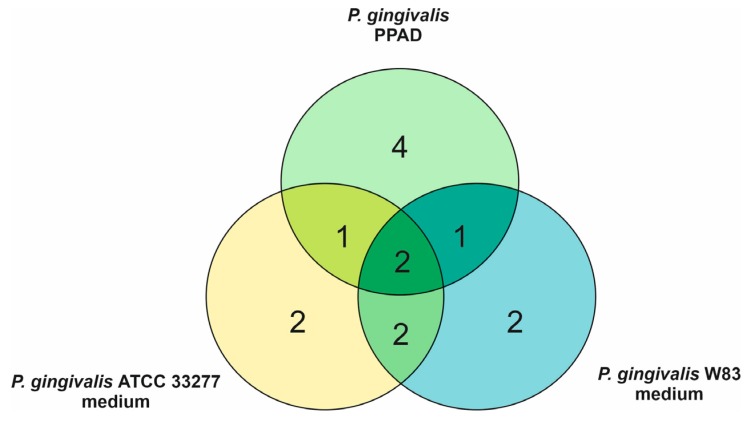
Venn diagram indicating overlapping and specific *C. albicans* proteins identified as citrullinated after treatment of the fungal cells with culture supernatants collected after *P. gingivalis* growth and with *P. gingivalis* PPAD.

The effects of purified *P. gingivalis* PPAD on the *C. albicans* surface-localized proteins were tested next. However, HRgpA was added to the PPAD preparation used at a low concentration so that the peptide fragments with C-terminal arginine residues prone to citrullination could be generated. A list of modified fungal proteins including their mass spectrometric identification criteria is presented in Table S3 (Supplementary File 3). Eight surface-exposed *C. albicans* proteins were indicated as citrullinated, including Eno1, Mp65, and Eng1 that were shown to be modified after fungal growth in bacterial supernatants (Table S2) and in mixed biofilms in our previous report [16]. In addition, the citrullination of Tdh3 was identified in our present analysis under both conditions used. In the case of alcohol dehydrogenase 1 (Adh1), citrullination was detected after treatment with PPAD and in mixed biofilm in our earlier study [16], whereas the citrullination of agglutinin-like sequence protein 3 (Als3), 1,3-beta-glucanosyltransferase Pga4 and pH-responsive protein 1 was detected only after treatment with PPAD. Quantitatively, the similarities and differences in the approaches used, namely the treatment of *C. albicans* cells with culture supernatants or with purified PPAD, with respect to the number of fungal proteins identified as citrullinated are shown in Figure 3.

**Figure 4:**
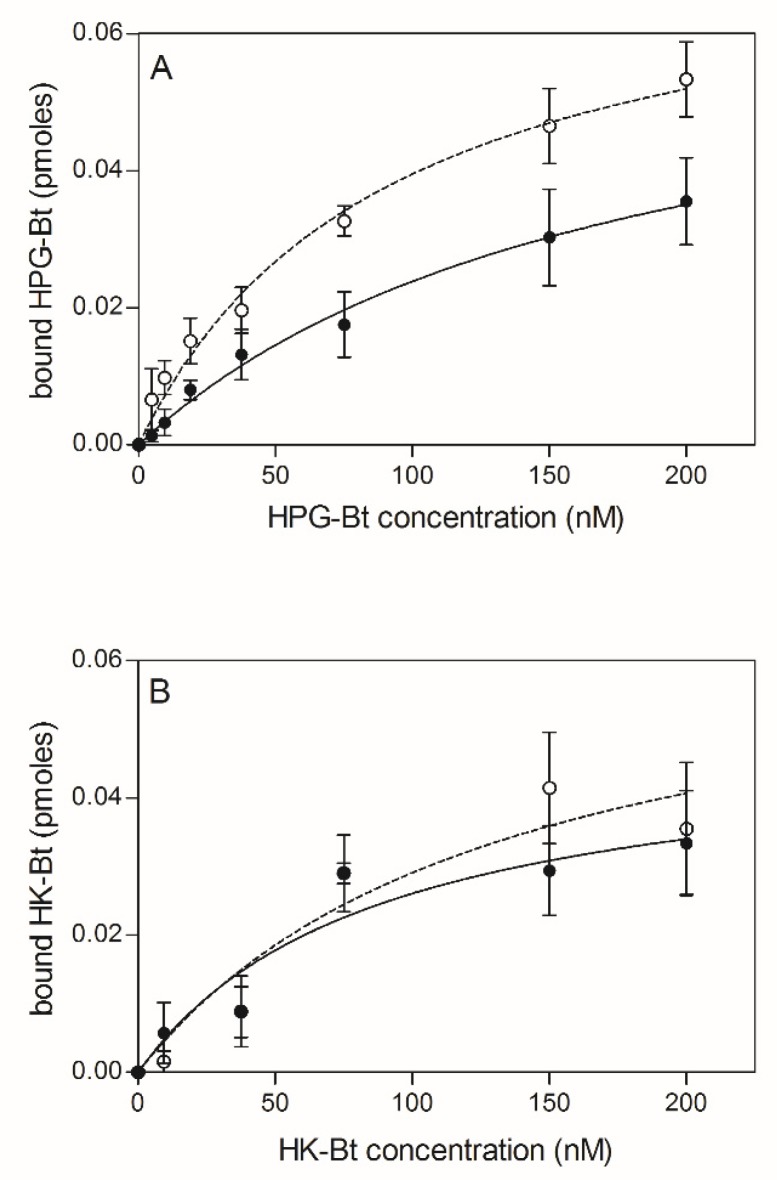
Binding of biotinylated human plasma proteins HPG-Bt (**A**) and HK-Bt (**B**) to *C. albicans* hyphae treated with *P. gingivalis* PPAD. The solid line and closed symbols refer to *C. albicans* cells incubated with 0.1 µM PPAD for 2 h. The dashed line and open symbols refer to unmodified fungal cells. Data points represent the mean values ± SEM.

The effects of the citrullination of fungal proteins on HPG and HK binding was next tested using *C. albicans* cells that had undergone surface modification by PPAD (Figure 4).

Similarly, to the previous instance, the bound HPG-Bt level seemed to depend on PPAD activity because the binding of this human protein was noticeably lower for citrullinated *C. albicans* cells than for their non-modified counterparts. In contrast, the binding of HK-Bt appeared to be independent of PPAD activity as the difference between HK-binding by PPAD-treated and untreated cells was not significant.

### 2.2. Effects of HPG and HK Citrullination on Their Binding to C. albicans Hyphae

**Figure 5:**
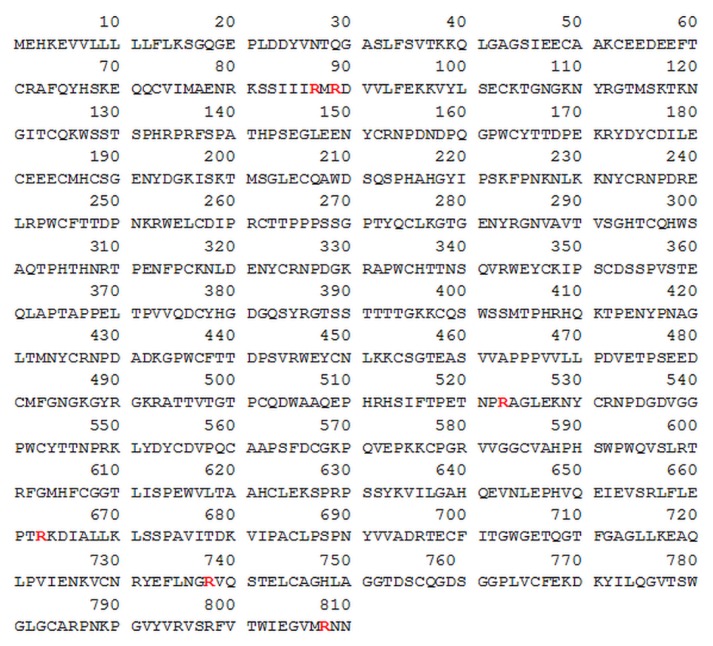
HPG sequence with arginine residues denoted in red text identified by LC-MS/MS as putatively modified by PPAD. The HPG sequence was obtained from UniProtKB database - P00747 (PLMN_HUMAN) record [41].

After investigating the impact of bacterial secretory proteins on fungal surface proteins and how their citrullination affects the binding to human plasma proteins, the adhesion of citrullinated human HPG and HK to unmodified *C. albicans* hyphae was subsequently tested. Several arginine residues were identified as potential sites on both proteins where modification by PPAD could occur (Figure 5; Figure 8). The reactions were carried out in the presence of HRgpA at an enzyme:substrate molar ratio of 1:100 to effectively expose the C-terminal arginine residues. Identification details are presented in Table S4 for HPG (Supplementary File 4) and Table S5 for HK (Supplementary File 5).

In the case of HPG, six arginine residues that can be modified to citrulline after incubation with the bacterial enzymes HRgpA and PPAD were identified using mass spectrometry. These were R87, R89 located near the N-terminus of HPG within the activation peptide, R523 in the kringle 5 domain and the R663, R738 and R808 residues in close proximity to the plasmin active site and protein C-terminus. Incubation of HPG only with PPAD, with additional inhibition of gingipain activity, did not result in efficient citrullination.

**Figure 6:**
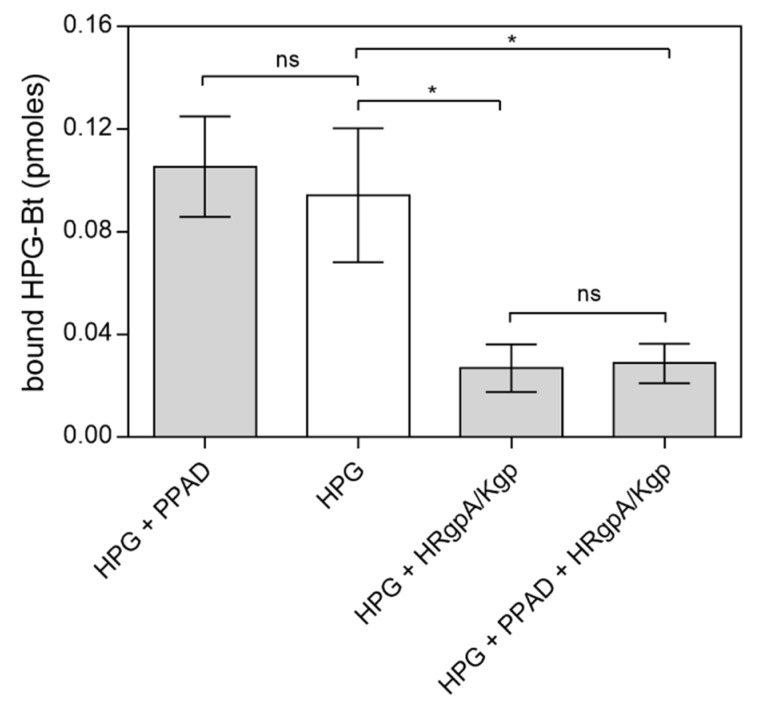
Binding of citrullinated HPG to *C. albicans* hyphae. A total of 150 nM HPG-Bt was incubated with 50 nM PPAD with or without the gingipains HRgpA and Kgp (enzyme:substrate molar ratio of 1:100) for 18 h at 37 °C and then applied for 1.5 h to microplate wells containing 5 × 10^5^ *C. albicans* hyphae. The bound biotinylated protein was quantified using the SA-HRP/TMB detection system. Untreated HPG served as a control. Bars represent the mean values ± SEM. Statistical analysis was performed by one-way ANOVA with Tukey’s multiple comparisons test (*p* < 0.05) using GraphPad Prism software. Statistical significance levels are indicated: * *p*< 0.05; ns, non-significant.

The binding of citrullinated HPG to the surface of fungal cells that were not subjected to modification indicated no significant effect of PPAD modification of human proteins on this phenomenon (Figure 6). If purified PPAD alone was used, the level of HPG binding was at the same level as the control sample. Only the addition of HRgpA and Kgp to HPG at the enzyme:substrate molar ratio of 1:100 caused any significant reduction in the level of HPG binding. However, the additional presence of PPAD in this mixture also resulted in no change in the adhesion level. During the contact between human or bacterial proteins with fungal cells, gingipain inhibitors were used to prevent further modifications of the *C. albicans* surface proteins.

**Figure 7:**
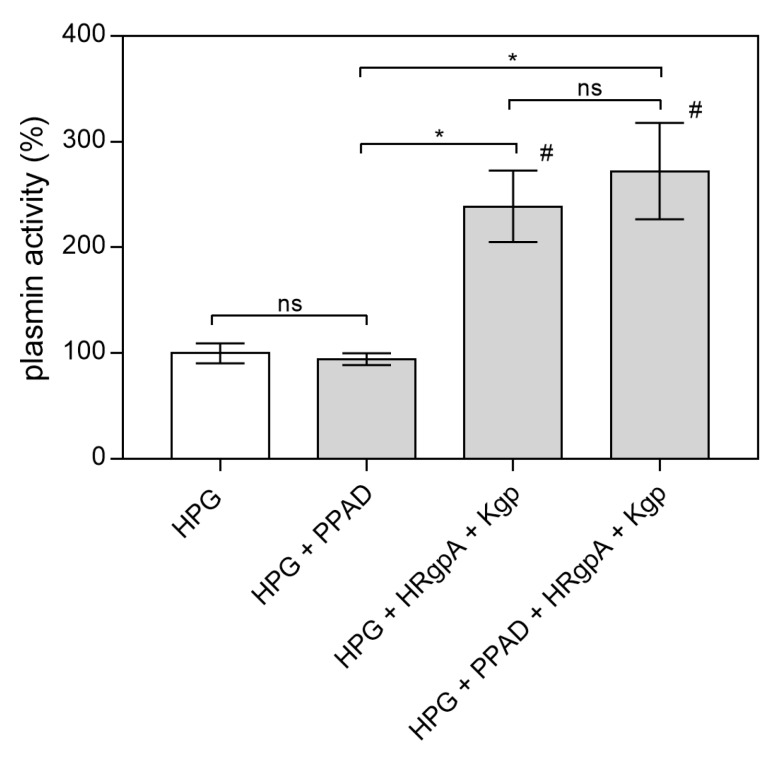
Conversion of HPG into active plasmin by HRgpA and Kgp in the presence of PPAD. A total of 300 nM HPG was incubated with 3 nM HRgpA and Kgp and 100 nM PPAD for 2 h at 37 °C. The gingipain inhibitors KYT-1 and KYT-36 were then added at a final concentration of 1 µM and the resulting plasmin activity in the presence of plasmin substrate was followed by monitoring the absorption of the released p-nitroaniline at 405 nm. Bars represent the mean values ± SEM. Untreated HPG served as a control. Statistical analysis was performed by one-way ANOVA with Tukey’s multiple comparisons test (*p* < 0.05) using GraphPad Prism. Statistical significance levels are indicated: * *p*< 0.05; ns, non-significant; # *p* < 0.05 vs. control.

The main function of HPG is proteolytic activity after its conversion to plasmin by plasminogen activators such as urokinase (uPA), tissue plasminogen activator (tPA) or streptokinase [42]. The complex of RgpA and Kgp has also been shown recently to activate the urokinase plasminogen activator system and form plasmin from HPG [37]. Hence, it was reasonable to test whether the citrullination of HPG in the presence of these gingipains may also affect this activation process. As shown in Figure 7, the modification of HPG by PPAD in the presence of HRgpA and Kgp had no effect on HPG activation to plasmin. After the modification of HPG, the gingipain inhibitors KYT-1 and KYT-36 were added to the reaction mixture to avoid hydrolysis of the plasmin substrate by these bacterial enzymes and to observe only the activity of the resulting plasmin.

**Figure 8:**
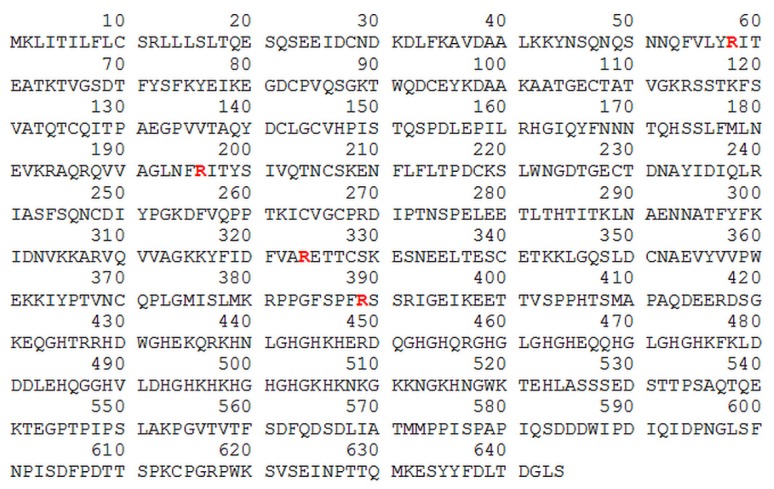
HK sequence, with arginine residues labelled in red text, identified by LC-MS/MS and as putatively modified by PPAD. The human HK sequence was obtained from the UniProtKB database - P01042 (KNG1_HUMAN) record [41].

A similar analysis of HK modifications revealed that four arginine residues could have been converted into citrulline after HK exposure to PPAD and the bacterial proteolytic enzyme, HRgpA, as shown in Figure 8 and Table S5.

Three of these arginines are located within the HK heavy chain, namely R58, R196, and R324, whereas the fourth, R389 is located at the C-terminus of the kinins, the sequence of which is located in domain 4. The incubation of HK alone with PPAD without gingipain activity did not allow citrullination to occur efficiently.

**Figure 9:**
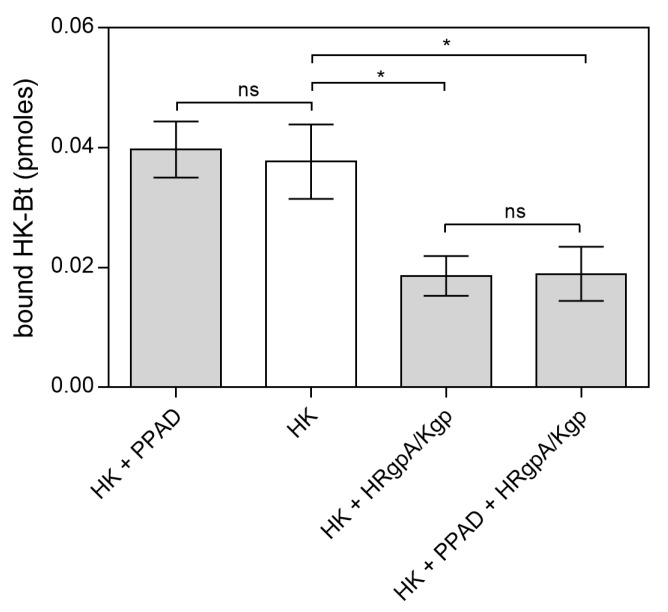
Binding of citrullinated HK to *C. albicans* hyphae. A total of 150 nM HK-Bt was incubated with 50 nM PPAD with or without the gingipains HRgpA and Kgp with an enzyme:substrate molar ratio of 1:100 for 18 h at 37 °C. The bound biotinylated protein was quantified using the SA-HRP/TMB detection system. Bars represent the mean values ± SEM. Untreated HK served as a control. Statistical analysis was performed by one-way ANOVA with Tukey’s multiple comparisons test (*p* < 0.05) using GraphPad Prism. Statistical significance levels are indicated: * *p*< 0.05; ns, non-significant.

**Figure 10:**
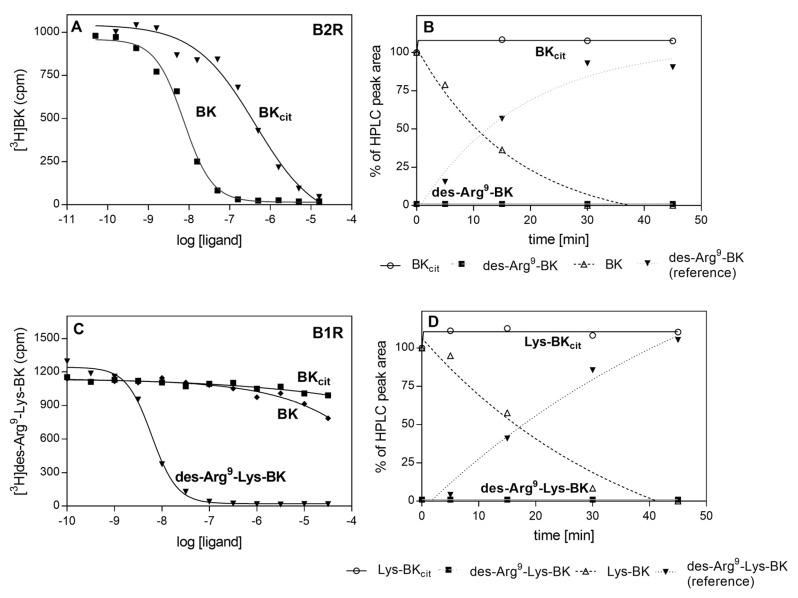
Binding of citrullinated BK to B2R (**A**) or B1R (**C**) and the degradation of BK_cit_ (**B**) and Lys-BK_cit_ (**D**) by CPM. Monolayers of HEK293 cells that overexpressed human B2R or B1R were incubated with a mixture of 2 nM [^3^H]BK with BK and BK_cit_ (**A**) or 1 nM [^3^H]des-Arg9-Lys-BK with des-Arg^9^-Lys-BK, BK and BK_cit_ (**C**). The competitors were added at a concentration range of 0.05 nM–50 µM. For the degradation assay, both BK_cit_ (**B**), Lys-BK_cit_ (**D**), and their reference compounds, were incubated with CPM at a ratio 1:50,000 in 50 mM HEPES buffer, pH 7.0 at 37 °C. Products of the enzymatic reactions formed in the different time intervals were separated by HPLC.

Similarly, to the binding of citrullinated HPG to *C. albicans* cells, the binding of PPAD-treated HK-Bt to the fungal cell surface was unchanged (Figure 9). Although the presence of HRgpA and Kgp significantly reduced the HK binding properties, the additional citrullination of HK after its proteolytic treatment did not significantly affect the amount of HK bound to the fungal cells. The most important function of HK is the release of the vasoactive and pro-inflammatory peptides, bradykinin (BK) and Lys-bradykinin (Lys-BK), which are generated from this protein by the kallikreins or via the actions of microbial proteases [43,44,45,46]. We thus further investigated the effects of citrullination of the C-terminal arginine residue in the kinins on their interaction with specific cellular receptors (Figure 10).

Kinins exert their biological effect via the stimulation of two major types of kinin receptors, B2 and B1 (B2R and B1R, respectively). The constitutively expressed and ubiquitous B2R is stimulated by BK and Lys-BK, whereas B1R, the expression of which is induced by proinflammatory cytokines, responds to kinins deprived of C-terminal Arg (des-Arg^9^-BK, des-Arg^9^-Lys-BK). Agonist interactions with kinin receptors result in the stimulation of angiogenesis, cell proliferation, increased vascular permeability and enhanced cytokine expression in different cells [47]. To test the effects of kinin citrullination on the peptide affinity to these receptors, kinin derivatives with C-terminal citrulline (BK_cit_) were tested for binding to B2R using a radioreceptor competitive assay. In this assay, the modified kinins competed with tritium-labeled BK for binding sites located on the HEK293 cells with overexpressed B2 receptor. BK_cit_ showed a significantly lower affinity (10^−7^ M vs. 10^−9^ M) for the B2 receptor compared to BK, the physiological agonist.

In vivo, the kinins are rapidly degraded by different kininases in the blood and other tissues [48]. The most important of these include carboxypeptidase M (CPM) that generates des-Arg^9^-BK, an agonist kinin for B1 receptors. To test whether citrullination can influence the degradation of kinins by this enzyme, BK_cit_ and Lys-BK_cit_ were subjected to CPM action and compared with the processing of non-modified kinins. The products of the enzymatic reaction were separated and identified using HPLC. This analysis revealed that the modification of kinin peptides prevents the formation of B1R agonists (Figure 10B,D). Additionally, a total loss of affinity to B1R was observed for BK_cit_ in the competition test with tritium labeled des-Arg^9^-Lys-BK bound to B1R-overexpressing HEK293 cells.

## 3. Discussion

Pathogenic microorganisms use various mechanisms to increase their virulence potential and successfully invade a host organism whilst avoiding the host immune response. This includes the modulation of important host plasma homeostatic systems such as the coagulation and fibrinolysis systems, the complement system, and the kinin generation system (also known as the contact system) [45,46,49,50,51,52]. One of the mechanisms that facilitates the interference by pathogens of plasma homeostatic systems is the binding of their individual components to the surfaces of microbial cells, which contributes to the local increase in their concentration and further processing by proteases, or to the insidious takeover of these components and their exclusion from the host systems [53,54]. Such abilities to entrain plasma cascades and thus evade host innate immunity were previously described for the opportunistic pathogen *C. albicans* [55,56]. A number of *C. albicans* surface-exposed proteins responsible for binding of human HPG or HK have been identified to date [57,58,59,60]. The consequence of HK binding may be the production of biologically active kinins capable to interact with B2 and B1 receptors, and also to stimulate human cells to produce proinflammatory cytokines [45,46,61]. However, the gathering of the plasma contact system components at the surface of *C. albicans* cells, followed by their activation, is considered to be a double-edge sword for the host. Whilst enabling stimulation of innate immunity and the influx of immune cells to the site of infection, this process also causes a leakage of plasma with nutrients to the infectious foci and opens a further path to the outflow and spread of microorganisms with the bloodstream [62]. 

The network of interactions between a host and fungal pathogen becomes even more complex if the fungal cells are accompanied by other pathogenic microorganisms at the site of infection. This occurs in the oral cavity―in subgingival biofilm, in the gingival pockets or root canal system―where *C. albicans* might form a mixed-species biofilm together with different bacteria, including strict anaerobes like *P. gingivalis*, providing them protection under aerobic conditions, and also during aggressive and chronic periodontitis [63,64,65]. It was indicated in our previous study that the activity of citrullinating bacterial virulence factor―PPAD―had a certain importance during the formation of dual-species bacterial-fungal biofilm [16]. The modifications that PPAD introduces to diverse molecules play a role not only in relation to the functionality of bacterial proteins, and modulation of biofilm development, but also in the interactions with host proteins and systems [25,66,67,68]. Under the conditions used in this study citrullination of such *C. albicans* proteins as glucan 1,3-beta-glucosidase Bgl2, elongation factor 1-alpha 1, Tdh3, heat shock proteins Ssa1 and Ssa2, yeast-form wall protein 1, plasma membrane ATPase 1, agglutinin-like sequence protein 3, 1,3-beta-glucanosyltransferase Pga4 and pH-responsive protein 1 was newly identified, whereas Eno1, Mp65, Adh1 and Eng1 were previously identified to be citrullinated [16]. Our current study findings demonstrate that the modifications of the surface proteins of *C. albicans* by bacterial enzymes, mainly PPAD and gingipains, can affect the interaction of fungal cells with HPG, reducing the level of its binding to the fungal cell surface. Among the citrullinated candidial proteins identified in this study were those indicated as HPG binding proteins in other reports, including Eno1, Adh1 and Tdh3 [38,57]. Hence, it is possible that citrullination may have an impact on the adhesion of HPG to *C. albicans*. Although in the case of enolase, frequently indicated as a major HPG-binding microbial protein, the identified modification of Arg333 is distant from the HPG-binding fragments located near its C-terminus containing the lysine-dependent binding site, its location in a higher-order structure may influence the enolase exposure at the fungal cell surface [53,69]. Furthermore, the effect of these modifications can be complex and depend on the interaction of many HPG-binding proteins present at the surface of the fungal cells and modified by PPAD, given that the detailed molecular bases of this phenomenon are currently not fully recognized for *C. albicans* proteins. On the other hand, citrullination within the HPG molecule fully retains its binding capacity for unmodified fungal surfaces, unlike proteolysis carried out by gingipains that significantly reduces the level of binding of this human protein to *C. albicans* hyphae. Interestingly, human alpha-enolase was also previously indicated as a protein citrullinated by *P. gingivalis* PPAD [22].

Importantly, despite the decrease in the level of binding, citrullination did not affect the conversion of HPG into plasmin. It has been shown previously that gingipains that might be present in such a mixed biofilm can convert HPG to plasmin [37,70], and therefore that this putative virulence mechanism based on the adsorption of HPG to fungal cells and its further activation can still be preserved, whilst some modulation of this system through the action of PPAD is possible.

Another important issue to recognize is the impact of citrullination on the process of kinin production at the site of infection by both microorganisms. During periodontitis, pain, redness and swelling develop within the infected tissue of the gums, triggered mainly by the kinins― potent mediators of inflammation, vasodilation and vascular permeability [29,71]. The local activation of the contact system at the infection foci might be beneficial for *P. gingivalis* by facilitating the dispersal of bacterial cells within the host organism [72]. As described in a previous report [73], these bacteria have the ability to bind HK and other components of the contact system on the cell surface mainly through the surface-exposed gingipains, thereby facilitating the production of kinins as a result of this adsorption. This phenomenon has been also described in detail for *C. albicans,* and several HK-binding proteins present at the surface of the fungal cells have been indicated [59,60], some of which have been identified by our present analysis as susceptible to citrullination by PPAD, including Als3 and Eno1. However, these modifications did not affect the level of HK binding to the modified fungal surface, nor did HK citrullination play a role in this process, unlike the gingipains which significantly reduced the binding of HK to fungal cells.

It should be noted that the C-terminal arginine residue of the kinin molecule was identified by our present analysis as being citrullinated by PPAD, as demonstrated also in earlier reports [19,23]. It is possible that such modifications can occur at the site of infection and during the formation of the mixed biofilm, where both the bacterial and fungal proteases are capable of generating kinins from HK. Kinin production on a local scale might be favourable for pathogens and the impact of citrullination on the interaction of kinins with their cellular receptors, through which they exert their biological effect, was therefore investigated in our present analysis. It was noteworthy from our present findings that the citrullination of the C-terminal Arg residue affects the binding of kinin to the B2 receptor type. The positive charge of the guanidine group in the unmodified C-terminal Arg residue compensates for the negative charge of its carboxyl group, thereby allowing the interaction with the B2 receptor [74]. Hence, citrullination can abolish this compensation effect, and as a consequence cause a significant decrease in the affinity for this receptor. In addition, a β-turn formed by the C-terminal Arg residue and Ser6 is critical for the interaction with B2R [47].

The native kinins are susceptible to the action of carboxypeptidase M, a regulatory enzyme that specifically hydrolyzes peptide bonds at the site of basic residues such as Lys and Arg [75]. The S1’ pocket of CPM is particularly adapted for P1’-Arg [76]. Hence, altering the charge of amino acid residues from positive to uncharged protects against proteolysis and results in an inability to remove the C-terminal residue from citrullinated BK. This results in the inhibition of des-Arg-kinin formation, the B1-type receptor agonist. A similar effect is observed during the modification of the C-terminal Arg of epithelial growth factor (EGF) by PPAD, which destroys its interaction with the EGF receptor (EGFR). In this case, citrullination presumably stabilizes the C-terminal alpha-helix through additional hydrophobic interactions. The modification prevents the maintenance of unorganized EFG structures, which is essential for the interaction with EGFR [77]. Likewise, the modification of the C-terminal Arg of the complement component C5a reduces its chemotactic activity [78]. Partial inhibition or slowing down of the action of the kinins by their citrullination could play a pivotal role in the regulation of inflammation by pathogens that invade the host. 

The interplay between bacterial and fungal virulence factors in a mixed biofilm formed at the infection foci, including the joint action of *P. gingivalis* enzymes―gingipains and PPAD―in the process of citrullination of human proteins―HPG and HK―that adhere to *C. albicans* cell surface, may be an important element of the pathogenesis mechanisms of these microbes. This involves a reversal of host defense mechanisms to the advantage of these invading microorganisms, and additional benefits of coexisting in a biofilm with other microbes.

## 4. Materials and Methods

### 4.1. Growth Conditions for Bacterial and Fungal Cultures

*P. gingivalis* wild-type strain W83 (ATCC^®^ BAA-308^TM^) from American Type Culture Collection (Manassas, VA, USA) and the mutant strain lacking the *ppad* gene (*Δppad* (W83)), obtained as described previously [22], were grown on TSBY agar plates (BTL, Lodz, Poland) supplemented with 5% v/v sheep blood and in liquid TSBY medium (50 mL) complemented with 250 mg/L L-cysteine-HCl, 0.5 mg/L vitamin K, 5 mg/L hemin (Sigma, St. Louis, MO, USA) at 37 °C in an anaerobic chamber (90% N_2_, 5% CO_2_, 5% H_2_) for 24 h. *P. gingivalis* wild-type strain 2561 (ATCC^®^ 33277™) and the mutant strain (*Δppad* (ATCC 33277)) deprived of the *ppad* gene as described previously by Gawron et al., 2014 [79], were grown on BHI blood agar plates (BD Company, Franklin Lakes, NJ, USA) and in 50 mL of BHI broth supplemented with 5 mg/L hemin and 0.5 mg/L vitamin K in an anaerobic chamber for 24 h. The media for the mutant strains were additionally supplemented with erythromycin (5 mg/L). Prior to use in the experiment, bacterial cells were discarded by double centrifugation of the whole culture (5000 × g, 30 min) and post-culture supernatants were collected and stored as frozen in −20 °C for further applications.

*C. albicans* strain 3147 (ATCC^®^ 10231^TM^) was purchased from American Type Culture Collection. Fungal cells were cultured at 30 °C for 16 h in 20 mL of liquid YPD medium (1% yeast extract, 2% soybean peptone and 2% glucose) (Sigma), then harvested by centrifugation (3000 rpm, 3 min) and washed twice with 1 mL of sterile phosphate-buffered saline (PBS), pH 7.4 (Biowest, Nuaillé, France). The optical densities at 600 nm were then measured to estimate the number of fungal cells. To subsequently induce hyphae formation, *C. albicans* cells were further cultured in RPMI 1640 medium for 18 h at 37 °C with shaking (170 rpm) in flasks (5 × 10^8^ cells per 20 mL) or in 100 µL of the same medium (5 × 10^6^ cells per well) in the wells of MaxiSorp 96-well microtiter plates (Nunc, Roskilde, Denmark).

### 4.2. Biotinylation of Human Proteins

For the biotinylation of human proteins, 100 µg aliquots of HPG or HK proteins (Enzyme Research Laboratories, South Bend, IN, USA) were incubated for 4 h at 4 °C in 250 µL of 0.1 M NaHCO_3_ buffer with 2 µL of biotin N-hydroxysuccinimide ester (NHS-biotin; Sigma) dissolved in dimethylformamide (1 mg/100 µL). The proteins were then dialyzed against PBS buffer, pH 7.4 for 48 h.

### 4.3. Modification of C. albicans Surface Proteins by Bacterial Enzymes

#### 4.3.1. Identification of Proteins Secreted by *P. gingivalis* into the Culture Medium

Aliquots (2 mL) of growth medium collected from cultures of *P. gingivalis* strains ATCC 33277, *∆ppad* (ATCC 33277), W83 and *Δppad* (W83) were lyophilized in an Alpha 1-2 lyophilizer (Martin Christ, Osterode am Harz, Germany) and then dissolved in 100 µL of ultra-pure water to achieve a 20-fold concentration. Three biological replicates were prepared. Samples for electrophoresis were prepared by mixing 15 μL of concentrated supernatant with 15 μL of loading buffer for subsequent SDS-PAGE electrophoresis followed by incubation at 95 °C for 5 min. After electrophoretic separation using the Laemmli system [80], protein detection was carried out with Coomassie Brilliant Blue G-250 staining and specific protein bands were excised from the gel. These gel samples were subjected to tryptic digestion followed by protein identification with LC-MS/MS using the Dionex UltiMate 3000 UHPLC system (Dionex, Carlsbad, CA) and the HCTUltra ETDII ion-trap mass spectrometer equipped with an electrospray ionization ion source (Bruker, Bremen, Germany), as described previously [81]. Protein identification was then performed using a SwissProt protein database search (560,118 sequences for all entries, including 336,487 sequences for bacterial proteins) with an in-house Mascot server (v.2.3.0, Matrix Science, London, UK).

#### 4.3.2. Growth of *C. albicans* Cells in Media Collected after Culture of *P. gingivalis*

*C. albicans* cells were first aerobically cultured in 20 mL of YPD medium for 16 h at 30 °C with shaking (170 rpm), and a 20 μL aliquot of the cell suspension was transferred to 20 mL of medium collected after 24 h of growth of *P. gingivalis* strains ATCC 33277, *Δppad* (ATCC 33277), W83, *Δppad* (W83) or to 20 mL of BHI or TSBY media, and further incubated for 18 h at 37 °C with shaking (170 rpm) under normoxia or anoxia. Anaerobic conditions were generated using anaerobic chambers, i.e., a GENbox jar with GENbox anaer generator (bioMérieux S.A., Marcy l’Etoile, France).

#### 4.3.3. Incubation of *C. albicans* Cells with PPAD from *P. gingivalis*

*P. gingivalis* PPAD was purified as described previously in detail by Goulas et al., 2015 [23]. Gingipains HRgpA and Kgp were purified from the post-culture medium of *P. gingivalis* HG66 strain using gel filtration and lysine- or arginine-Sepharose chromatography [82]. *C. albicans* cells (5 × 10^8^) were grown in RPMI 1640 medium for 18 h at 37 °C in MaxiSorp 96-well microtiter plates (Nunc) to which 100 µL solution of 0.1 µM PPAD, preactivated for 20 min in 10 mM HEPES buffer with 150 mM NaCl, 5 mM CaCl_2_, 10 mM cysteine, pH 7.5, was added. The enzymatic reaction was then further carried out for 2 h at 37 °C and the reaction mixture was supplemented with 0.015 nM HRgpA and Kgp to enable the C-terminal arginine residues in the proteins available for PPAD modification.

### 4.4. Cell Surface Shaving with Trypsin and Identification of C. albicans Proteins Modified by PPAD

Cell surface shaving with trypsin was performed as described earlier [16,83] and three biological replicates were prepared. Briefly, 5 × 10^8^ *C. albicans* cells grown in bacterial culture media or incubated with the purified PPAD as described above were washed three times with 1 mL of 25 mM NH_4_HCO_3_ with centrifugation for 4 min at 3000 rpm. Next, 100 μL aliquots of 5 mM dithiothreitol in 25 mM NH_4_HCO_3_ and 10 μL of sequencing grade modified trypsin (200 ng/μL) (Promega, Madison, WI, USA) were then added to the cells, followed by incubation for 10 min at 37 °C. After this time, the cells were centrifuged for 5 min at 5000 rpm, and the supernatant was filtered through 0.22 µm pores (Millex-GV, Sigma) to remove cell debris and impurities. The samples prepared in this way were subjected to further trypsin hydrolysis overnight at 37 °C. In the next step, trifluoroacetic acid (TFA) was added to a concentration of 0.1% to stop the enzymatic reaction. After incubation for 15 min at 4 °C samples were centrifuged for 15 min at 12,000 rpm, evaporated using a Centrivap concentrator 5415C (Labconco, Kansas City, MO, USA) and frozen for further analysis. The samples were then dissolved in 110 μL of 10% acetonitrile with 0.1% formic acid and analyzed using LC-MS/MS. The obtained results were analyzed using the Mascot server and SwissProt database (560,118 sequences for all entries, including 34,524 sequences for fungal proteins), taking into account variable modifications of arginine (citrullination), asparagine or glutamine (deamidation) residues, both leading to the additional mass of 1 Da. To avoid false positives corresponding to deamidation, the MS/MS fragmentation spectra for peptides indicated as citrullinated after automatic search, were manually reviewed by the verification of sequence-specific MS/MS fragment ions to confirm the identification of citrullination of C-terminal arginine resulting in the increase in mass exactly for this particular residue. It was also considered that the peptide properly indicated as citrullinated was assigned a significantly higher identification score in comparison with the other suggested sequences. Peptides in which specific arginine residues had been modified to citrulline in all three biological replicates were used for subsequent analysis.

### 4.5. Analysis of the Modifications of Human Proteins by Bacterial PPAD

A total of 50 nM PPAD was activated for 20 min at 37 °C in 10 mM HEPES buffer with 150 mM NaCl, 5 mM CaCl_2_, 10 mM cysteine, pH 7.5, and with the addition of gingipain HRgpA and Kgp at a concentration of 1.5 nM. To investigate the citrullination of human proteins, aliquots of HPG or HK were added to PPAD solution to the final concentration of 150 nM and further incubated for 2 h at 37 °C. Trypsin (2µL; Promega) was then added and the samples were incubated for 4 h at 37 °C. TFA was then added to the sample at a final concentration of 0.1% and a further incubation for 15 min on ice was carried out, followed by centrifugation at 4 °C for 15 min at 10,000 rpm. The obtained peptides were desalted with C18 tips (Pierce^®^ C18 Tips, Thermo Scientific, Waltham, MA, USA) and analysis with LC-MS/MS was performed to indicate the modifications. Citrullination of arginine residues and deamidation of asparagine and glutamine residues, both resulting in the mass increase by 1 Da, were identified during the analysis with the Mascot server and SwissProt database (560,118 sequences for all entries, including 20,352 sequences for human proteins). Similar to that described above, the correct assignment of citrullination to individual arginine residues, instead of peptide deamidation, was confirmed with manual verification of sequence specific MS/MS fragment ions and the ion scores for listed matching sequences. Moreover, the retention time of citrullinated peptide during the separation with reversed-phase liquid chromatography was prolonged in contrast to the unmodified corresponding peptide, as a result of the loss of arginine positive charge [84,85].

### 4.6. Binding of HPG-Bt and HK-Bt to C. albicans Cells Pre-Treated with Bacterial Enzymes

Aliquots of 5 × 10^5^ *C. albicans* cells per well of a MaxiSorp microplate (Nunc) were grown in 100 µL of RPMI 1640 medium at 37 °C for 18 h. The cells were then washed three times with 200 µL of PBS and incubated with (i) 50 µL of 0.1 µM PPAD with 0.015 nM HRgpA and Kgp for 2 h at 37 °C or (ii) 100 µL of media collected after growth of *P. gingivalis* strains ATCC 33277, *∆ppad* (ATCC 33277), W83, *Δppad* (W83) for the next 18 h at 37 °C. After washing out of the bacterial enzymes with PBS, the reactions in the wells were blocked using 300 µL of 3% bovine serum albumin (BSA) for 1 h at 37 °C. Then, 50 µL of each human protein solution in the concentration range from 3.125 nM to 200 nM were added to *C. albicans* cells for 1.5 h at 37 °C. Bound proteins were detected with the SA-HRP/TMB (streptavidin conjugated to horseradish peroxidase/3,3′,5,5′-tetramethylbenzidine) detection system and absorbance at 450 nm was measured with a Power Wave X Select microplate reader (BioTek Instruments, Winoosky, VT, USA). This experiment was performed in three independent replicates.

### 4.7. Binding of Citrullinated HPG and HK to C. albicans Cells

HPG or HK (150 nM) proteins were mixed with gingipain HRgpA and Kgp at the enzyme:substrate molar ratio of 1:100 and with 50 nM PPAD in 10 mM HEPES buffer with 150 mM NaCl, 5 mM CaCl_2_, 10 mM cysteine, pH 7.5, and incubated for 18 h at 37 °C. HK or HPG incubated with buffer only served as controls. Additionally, in the case of the samples prepared with PPAD enzyme alone, the gingipain inhibitors KYT-1 and KYT-36 (described by Kadowaki, 2004 [86]) were added at a final concentration of 1 µM to eliminate any unwanted proteolytic activity. After 18 h of incubation, gingipain inhibitors were added to all prepared samples to stop the proteolytic reaction. Then, 5 × 10^5^ of *C. albicans* cells in each well of a microplate were grown in RPMI 1640 medium for 18 h at 37 °C. The wells were washed three times after each step with 200 µL of PBS. The unoccupied well surfaces were blocked with 300 µL of 3% BSA for 1 h at 37 °C, and 50 µL aliquots of citrullinated proteins were added to the cells for 1.5 h at 37 °C. Bound proteins were detected using the SA-HRP/TMB system. The experiment was performed in three independent replicates.

### 4.8. Plasmin Activity Assay

The reaction mixture was prepared by incubation of 1.5 µM HPG, 0.5 µM PPAD and 15 nM HRgpA and Kgp in 10 mM HEPES buffer with 150 mM NaCl, 5 mM CaCl_2_, 10 mM cysteine, pH 7.5, for 2 h at 37 °C. Subsequently, 1 µM concentrations of the gingipain inhibitors KYT-1 and KYT-36 were added to prevent substrate hydrolysis by these enzymes. In the wells of a 96-well microplate (Sartsedt, Nümbrecht, Germany) 80 µL of 0.1 M TRIS buffer with 0.2 M NaCl, pH 7.8, was placed together with 0.2 mM substrate for plasmin, H-D-Ile-Phe-Lys-pNA (Bachem, Bubendorf, Switzerland), and 20 µL of reaction mixture prepared as described above, for further incubation at 37 °C. The amount of released p-nitroaniline was determined by an increase in the absorbance at 405 nm.

### 4.9. Competitive Radioreceptor Assay for Kinins

The competitive radioreceptor assay to assess kinin affinity to B2 or B1 receptors was performed as detailed previously [33]. Briefly, to 5 × 10^4^ of HEK293 cells with overexpressed kinin receptors and obtained using the Flp-In T-Rex-System (Invitrogen, Carlsbad, CA, USA) [87] and cultured in Dulbecco’s modified Eagle’s medium (DMEM) with a high-glucose concentration, 2 mM glutamine, and sodium pyruvate, a mixture of 2 nM [^3^H]-bradykinin (RPPGFSPFR) (Perkin-Elmer, Waltham, MA, USA), bradykinin, des-Arg^9^-Lys-bradykinin (KRPPGFSPF) (Bachem) or citrullinated bradykinin (RPPGFSPFCit, BK_cit_) (Chi Scientific, Maynard, MA, USA) at concentrations within a range of 0.05 nM – 50 μM was added for 1.5 h [88]. After washing out the unbound compounds, an incubation in 0.2 M acetate buffer with 0.5 M NaCl (pH 2.7) was used to dissociate the [^3^H]-labelled tracer peptides and the radioactivity was measured in 2.5 mL of Ultima Gold™ scintillation fluid (Perkin-Elmer) with a beta counter (Wallace 1412; LKB, Uppsala, Sweden).

### 4.10. Kinin Degradation by Carboxypeptidase M

Degradation assays were performed by incubating synthetic kinin derivatives (BK_cit_, Lys-BK_cit_) and their unmodified references with CPM (R&D Systems, Minneapolis, MN, USA) in 50 mM HEPES buffer, pH 7.0 at 37 °C at an enzyme:substrate weight ratio of 1:50,000. In time intervals of 5, 15, 30, and 45 min, the reaction was stopped with 1 µL of 100 µM 2-mercaptomethyl-3-guanidinoethylthiopropranoic acid (MGTA) (Calbiochem, San Diego, CA, USA). Then, 100 µL aliquots were then supplemented with 20 µL HCl and analyzed by HPLC with a reference to appropriate des-Arg-kinin standards. The modified peptides and degradation products were centrifuged (10,000 × *g*, 2 min) and analyzed using a Eurosil Bioselect 300-5 analytical HPLC column C18 (200 x 4 mm) equipped with a cartridge precolumn (Knauer, Berlin, Germany). Separations were performed at room temperature using a two-solvent gradient system (solvent A: 0.1% TFA, solvent B: 0.08% TFA in 80% acetonitrile) at a flow rate of 1 mL/min, in a linear 40 min gradient starting from 10% B with a slope of +1.0% solvent B/minute and with monitoring of the eluate at an absorbance of 215 nm.

## Supplementary Materials

Supplementary materials can be accessed at https://www.mdpi.com/1422-0067/21/7/2495/s1. Table S1. Mass spectrometric identification of proteins secreted into the growth medium by *P. gingivalis* strains W83, *Δ**ppad* (W83), ATCC 33277 and *Δ**ppad* (ATCC 33277). Table S2. *C. albicans* proteins citrullinated after incubation with culture supernatants collected after *P. gingivalis* growth. Table S3. *C. albicans* proteins citrullinated after incubation with *P. gingivalis* PPAD. Table S4. Peptides identified for HPG that had been citrullinated by bacterial PPAD in the presence of gingipains. A total of 150 nM human plasminogen was incubated with 50 nM PPAD and 1.5 nM HRgpA and Kgp for 18 h at 37 °C, and the peptides obtained after trypsin digestion were analyzed by LC-MS/MS. The theoretical mass in each case was calculated using PeptideMass software on the ExPASy server [89]. Table S5. Peptides identified for HK that had been citrullinated by bacterial PPAD in the presence of gingipains. A total of 150 nM HK was incubated with 50 nM PPAD and 1.5 nM HRgpA and Kgp for 18 h at 37 °C. The peptides subsequently obtained after trypsin digestion were analyzed by LC-MS/MS. The theoretical mass in each case was calculated using PeptideMass software on the ExPASy server [89].

## Abbreviations

BK	bradykinin
B1R	kinin receptor B1
B2R	kinin receptor B2
CPM	carboxypeptidase M
HK	high-molecular-mass kininogen
HK-Bt	biotinylated high-molecular-mass kininogen
HPG	plasminogen
HPG-Bt	biotinylated plasminogen
HPLC	high performance liquid chromatography
HRgpA	gingipain R
Kgp	gingipain K
LC-MS/MS	liquid chromatography coupled-tandem mass spectrometry
PPAD	peptidylarginine deiminase
SA-HRP/TMB	streptavidin conjugated to horseradish peroxidase/ 3,3′,5,5′-tetramethylbenzidine
SAPs	secreted aspartyl proteases
