# Supplementary material for: Peptidylarginine Deiminase of Porphyromonas gingivalis Modulates the Interactions between Candida albicans Biofilm and Human Plasminogen and High-Molecular-Mass Kininogen
Source: Int J Mol Sci. 2020 Apr 3;21(7):2495. doi: 10.3390/ijms21072495 (PMC7177930; doi:10.3390/ijms21072495)
Supplement: Supplementary file 1 [file ijms-21-02495-s001.zip › Supplementary Files revision/Supplementary File 1.pdf]

Table S1. Mass spectrometric identification of proteins secreted into the growth medium by *P. gingivalis* strains W83, *Δppad* (W83), ATCC 33277 and *Δppad* (ATCC 33277). Bacterial media were collected by centrifugation, concentrated 20 times by lyophilization, and prepared for SDS-PAGE electrophoresis. Proteins separated in these gels were stained with Coomassie Brilliant Blue G-250. To identify proteins secreted into the medium, liquid chromatography-coupled tandem mass spectrometry (LC-MS/MS) was used. The results from three independent experiments are presented (SC - sequence coverage).

***P. gingivalis* ATCC 33277**								
**Accession number**	**Protein name**	**Mass [kDa]**	**pI**	**Score**	**Matches**	**Sequences**	**emPAI**	**SC [%]**
B2RM93 (CPG1_PORG3)	Gingipain R1, *Porphyromonas gingivalis* (strain ATCC 33277 / DSM 20709 / CIP 103683 / JCM 12257 / NCTC 11834 / 2561)	186.463	4.97	195	17	13	0.11	8
				283	19	15	0.15	13
				224	12	11	0.10	8
B2RKJ1 (DHE2_PORG3)	NAD-specific glutamate dehydrogenase, *Porphyromonas gingivalis* (strain ATCC 33277 / DSM 20709 / CIP 103683 / JCM 12257 / NCTC 11834 / 2561)	49.623	5.98	127	8	8	0.26	18
				93	7	6	0.26	19
				89	6	6	0.26	16
B2RH54 (FIMA1_PORG3)	Major fimbrium subunit FimA type-1, *Porphyromonas gingivalis* (strain ATCC 33277 / DSM 20709 / CIP 103683 / JCM 12257 / NCTC 11834 / 2561)	41.569	5.84	120	7	4	0.40	17
				354	15	10	0.61	45
				487	21	11	0.97	47
B2RLK2 (KGP_PORG3)	Lys-gingipain, *Porphyromonas gingivalis* (strain ATCC 33277 / DSM 20709 / CIP 103683 / JCM 12257 / NCTC 11834 / 2561)	188.228	4.99	61	2	2	0.02	1
				63	3	3	0.02	2
				58	3	3	0.02	3
P72197 (KGP66_PORGN)	Lys-gingipain HG66, *Porphyromonas gingivalis*	187.741	4.97	202	16	14	0.17	9
				137	12	10	0.06	7
				243	11	11	0.10	8
Q9S3R8 (OMP40_PORGI)	Outer membrane protein 40, *Porphyromonas gingivalis* (strain ATCC BAA-308 / W83)	42.596	7.68	62	3	3	0.14	11
				52	2	2	0.07	6

Q9RQJ2 (PAD_PORGI)	Peptidylarginine deiminase, *Porphyromonas gingivalis* (strain ATCC BAA-308 / W83)	62.147	5.88	62	4	4	0.20	11
				73	8	7	0.10	16
				103	8	6	0.20	14
***P. gingivalis* Δ*ppad* (ATCC 33277)**								
**Accession number**	**Protein name**	**Mass [kDa]**	**pI**	**Score**	**Matches**	**Sequences**	**emPAI**	**SC %**
B2RM93 (CPG1_PORG3)	Gingipain R1, *Porphyromonas gingivalis* (strain ATCC 33277 / DSM 20709 / CIP 103683 / JCM 12257 / NCTC 11834 / 2561)	186.463	4.97	121	9	8	0.06	6
				107	10	8	0.05	6
				100	10	7	0.05	5
P95493 (CPG2_PORGI)	Gingipain R2, *Porphyromonas gingivalis* (strain ATCC BAA-308 / W83)	81.315	5.53	84	1	1	0.04	2
				52	5	5	0.11	9
				109	7	6	0.11	10
B2RKJ1 (DHE2_PORG3)	NAD-specific glutamate dehydrogenase, *Porphyromonas gingivalis* (strain ATCC 33277 / DSM 20709 / CIP 103683 / JCM 12257 / NCTC 11834 / 2561)	49.623	5.98	116	13	13	0.41	26
				94	4	3	0.19	11
				69	5	5	0.12	10
B2RH54 (FIMA1_PORG3)	Major fimbrium subunit FimA type-1, *Porphyromonas gingivalis* (strain ATCC 33277 / DSM 20709 / CIP 103683 / JCM 12257 / NCTC 11834 / 2561)	41.569	5.84	43	3	3	0.15	12
				56	4	4	0.15	14
				90	7	5	0.23	19
P72197 (KGP66_PORGN)	Lys-gingipain HG66, *Porphyromonas gingivalis*	187.741	4.97	54	3	3	0.02	2
				67	9	9	0.06	6
				131	9	9	0.08	7
P46071 (PRTH_PORGI)	Protease PrtH , *Porphyromonas gingivalis* (strain ATCC BAA-308 / W83)	111.251	8.58	54	2	2	0.03	2
				42	2	2	0.03	3
***P. gingivalis* W83**								
**Accession number**	**Protein name**	**Mass [kDa]**	**pI**	**Score**	**Matches**	**Sequences**	**emPAI**	**SC %**
B2RM93 (CPG1_PORG3)	Gingipain R1, *Porphyromonas gingivalis* (strain ATCC 33277 / DSM 20709 / CIP 103683 / JCM 12257 / NCTC 11834 / 2561)	186.463	4.97	369	29	26	0.28	18
				322	16	14	0.11	8
				297	19	16	0.17	10

B2RKU0 (CPG2_PORG3)	Gingipain R2, *Porphyromonas gingivalis* (strain ATCC 33277 / DSM 20709 / CIP 103683 / JCM 12257 / NCTC 11834 / 2561)	81.278	5.52	264	14	11	0.42	18
				249	11	9	0.15	16
				214	11	9	0.19	16
P95493 (CPG2_PORGI)	Gingipain R2, *Porphyromonas gingivalis* (strain ATCC BAA-308 / W83)	81.315	5.53	252	14	11	0.19	18
				245	9	7	0.15	9
				244	13	10	0.28	16
B2RKJ1 (DHE2_PORG3)	NAD-specific glutamate dehydrogenase, *Porphyromonas gingivalis* (strain ATCC 33277 / DSM 20709 / CIP 103683 / JCM 12257 / NCTC 11834 / 2561)	49.623	5.98	99	6	6	0.26	14
				98	7	5	0.33	14
				65	3	3	0.19	11
P0C935 (DPS_PORGI)	DNA protection during starvation protein, *Porphyromonas gingivalis* (strain ATCC BAA-308/ W83)	17.910	4.96	66	4	4	0.36	25
				43	3	3	0.17	18
P59915 (HAGA1_PORGI)	Hemagglutinin A, *Porphyromonas gingivalis* (strain ATCC BAA-308 / W83)	234.897	4.65	86	2	2	0.01	1
				103	9	7	0.05	3
				148	10	9	0.06	6
B2RLK2 (KGP_PORG3)	Lys-gingipain, *Porphyromonas gingivalis* (strain ATCC 33277 / DSM 20709 / CIP 103683 / JCM 12257 / NCTC 11834 / 2561)	188.228	4.99	248	8	7	0.08	6
				341	20	17	0.18	10
				159	14	14	0.10	10
P72197 (KGP66_PORGN)	Lys-gingipain HG66, *Porphyromonas gingivalis*	187.741	4.97	213	17	17	0.13	12
				342	21	18	0.20	10
				159	15	15	0.10	10
Q51817 (KGP83_PORGN)	Lys-gingipain W83, *Porphyromonas gingivalis*	188.784	5.03	242	10	9	0.08	18
				330	21	17	0.18	11
				269	23	20	0.16	14
Q9S3R8 (OMP40_PORGI)	Outer membrane protein 40, *Porphyromonas gingivalis* (strain ATCC BAA-308 / W83)	42.596	7.68	102	8	5	0.30	16
				41	6	6	0.07	16
				89	5	4	0.14	14
Q9S3R9 (OMP41_PORGI)	Outer membrane protein 41, *Porphyromonas gingivalis* (strain ATCC BAA-308 / W83)	43.511	8.61	72	2	2	0.07	9
				60	9	8	0.21	29
				42	3	3	0.07	10
Q9RQJ2 (PAD_PORGI)	Peptidylarginine deiminase, *Porphyromonas gingivalis* (strain ATCC BAA-308 / W83)	62.147	5.88	150	17	14	0.32	22
				269	19	13	0.58	24
				221	11	9	0.38	19
P46071 (PRTH_PORGI)	Protease PrtH, *Porphyromonas gingivalis* (strain ATCC BAA-308 / W83)	111.251	8.58	42	2	2	0.03	2
				74	10	9	0.08	10

				69	6	6	0.03	5
P25806 (TPR_PORGI)	Thiol protease, *Porphyromonas gingivalis* (strain ATCC BAA-308 / W83)	55.241	5.58	81	5	5	0.11	7
				69	6	6	0.11	15
***P. gingivalis* Δ*ppad* (W83)**								
**Accession number**	**Protein name**	**Mass [kDa]**	**pI**	**Score**	**Matches**	**Sequences**	**emPAI**	**SC %**
B2RM93 (CPG1_PORG3)	Gingipain R1, *Porphyromonas gingivalis* (strain ATCC 33277 / DSM 20709 / CIP 103683 / JCM 12257 / NCTC 11834 / 2561)	186.463	4.97	245	15	11	0.13	7
				306	17	13	0.15	7
				246	23	21	0.15	14
B2RKU0 (CPG2_PORG3)	Gingipain R2, *Porphyromonas gingivalis* (strain ATCC 33277 / DSM 20709/ CIP 103683 / JCM 12257 / NCTC 11834 / 2561)	81.278	5.52	71	3	3	0.04	6
				55	4	3	0.07	6
P95493 (CPG2_PORGI)	Gingipain R2, *Porphyromonas gingivalis* (strain ATCC BAA-308 / W83)	81.315	5.53	302	14	10	0.32	16
				249	11	8	0.23	12
				112	10	6	0.07	9
B2RKJ1 (DHE2_PORG3)	NAD-specific glutamate dehydrogenase, *Porphyromonas gingivalis* (strain ATCC 33277 / DSM 20709 / CIP 103683 / JCM 12257 / NCTC 11834 / 2561)	49.623	5.98	126	13	11	0.58	29
				168	8	6	0.26	15
				92	7	7	0.26	16
P72197 (KGP66_PORGN)	Lys-gingipain HG66, *Porphyromonas gingivalis*	187.741	4.97	324	20	17	0.22	11
				122	9	8	0.05	7
				140	12	11	0.08	8
Q51817 (KGP83_PORGN)	Lys-gingipain W83, *Porphyromonas gingivalis*	188.784	5.03	122	9	8	0.05	8
				361	24	18	0.24	13
				140	12	10	0.08	7
Q9S3R8 (OMP40_PORGI)	Outer membrane protein 40, *Porphyromonas gingivalis* (strain ATCC BAA-308 / W83)	42.596	7.68	100	3	2	0.14	7
				64	1	1	0.07	4
