# Supplementary material for: Peptidylarginine Deiminase of Porphyromonas gingivalis Modulates the Interactions between Candida albicans Biofilm and Human Plasminogen and High-Molecular-Mass Kininogen
Source: Int J Mol Sci. 2020 Apr 3;21(7):2495. doi: 10.3390/ijms21072495 (PMC7177930; doi:10.3390/ijms21072495)
Supplement: Supplementary file 1 [file ijms-21-02495-s001.zip › Supplementary Files revision/Supplementary File 2.pdf]

Table S2. ***C. albicans*** **proteins citrullinated after incubation with culture supernatants collected after** ***P. gingivalis*** **growth.**

$5x10^8$ of *C. albicans* cells grown in medium collected after culture of *P. gingivalis* strains ATCC 33277, *Δppad* (ATCC 33277), W83 and *Δppad* (W83), in aerobic or anaerobic conditions were then incubated with trypsin to hydrolyze surface-localized proteins. The obtained peptides were subjected to LC-MS/MS identification. The results from three independent experiments are presented (the asterisk denotes proteins identified as citrullinated for the first time in this study).

C. albicans **cells incubated in** ***P. gingivalis*** **ATCC 33277 medium**							
**Accession number**	***C. albicans*** **protein name**	**Peptide**	***P. gingivalis*** **strain**	**Observed m/z ratio (charge)**	**Calculated mass [Da]**	**Ion score**	
						**+ Ox**	**-Ox**
Q5AMT2 (BGL2_CANAL)*	Glucan 1,3-beta-glucosidase BGL2, *Candida albicans* (strain SC5314 / ATCC MYA-2876)	^{113}IFLVGSEALY**R**^{123}	—	634,4490 (+2)	1266,8834	82	26
			ATCC33277	634,9690 (+2)	1267,9234	69	
			Δ*ppad*	634,4400 (+2)	1266,8654	69	88
P0CY35 (EF1A1_CANAL)*	Elongation factor 1-alpha 1, *Candida albicans* (strain SC5314 / ATCC MYA-2876)	^{254}IGGIGTVPVG**R**^{264}	—	513,2960 (+2)	1024,5774	46	
			ATCC33277	513,8000 (+2)	1025,5854	33	
			Δ*ppad*	513,3000 (+2)	1024,5854	153	
Q5AIR7 (ENG1_CANAL)	Endo-1,3(4)-beta-glucanase 1, *Candida albicans* (strain SC5314 / ATCC MYA-2876)	^{932}DASNPSADDTYFPVS**R**^{947}	—	871,5000 (+2)	1740,9854	73	48
			ATCC33277	871,9000 (+2)	1741,7854	40	
			Δ*ppad*	871,5000 (+2)	1740,9854	73	41
Q92211 (G3P_CANAW)*	Glyceraldehyde-3-phosphate dehydrogenase, *Candida albicans* (strain WO-1)	^{234}VPTTDVSVVDLTV**R**^{247}	—	750,9000 (+2)	1499,7854	152	
			ATCC33277	751,4890 (+2)	1500,9634	60	
			Δ*ppad*	750,9000 (+2)<br>501,0000 (+3)	1499,9782	154	67
P41797 (HSP71_CANAL)*	Heat shock protein SSA1, *Candida albicans* (strain SC5314 / ATCC MYA-2876)	^{35}TTPSFVAFTDTE**R**^{47}	—	736,4370 (+2)	1470,8594		
			ATCC33277	736,9370 (+2)	1471,8594	31	
			Δ*ppad*	736,4000 (+2)	1470,7854	81	28
		^{158}DAGTIAGLNVL**R**^{169}	—	600,3550 (+2)	1198,6954	71	
			ATCC33277	600,9380 (+2)	1199,8614	83	

			Δ*ppad*	600,3950 (+2)	1198,7754	47	48
Q59XX2 (MP65_CANAL)	Cell surface mannoprotein MP65, *Candida albicans* (strain SC5314 / ATCC MYA-2876)	^{142}SESQIASEIAQLSGFDVI**R**^{160}	—	684,0000 (+3)<br>1025,5000 (+2)	2048,9854	152	
			ATCC33277	684,4210 (+3)<br>1026,1000 (+2)	2050,1854	76	59
			Δ*ppad*	1025,5820 (+2)<br>684,0930 (+3)	2049,1494	107	102
		^{311}NVLITETGWPS**R**^{322}	—	686,9000 (+2)	1371,7854	148	
			ATCC33277	687,4000 (+2)	1372,7854	65	
			Δ*ppad*	686,9650 (+2)	1371,9154	91	79
Q59Y31 (YWP1_CANAL)*	Yeast-form wall Protein 1, *Candida albicans* (strain SC5314 / ATCC MYA-2876)	^{77}YNTDI**R**^{82}	—	391,2690 (+2)	780,5234	77	22
			ATCC33277	391,7000 (+2)	781,3854	21	
			Δ*ppad*	391,2000 (+2)	780,3854	48	32
		^{87}VINVPA**R**^{93}	—	384,7910 (+2)	767,5674	46	27
			ATCC33277	385,2000 (+2)	768,3854	40	
			Δ*ppad*	384,8000 (+2)	767,5854	42	27
C. albicans **cells incubated in** ***P. gingivalis*** **W83 medium**							
**Accession number**	***C. albicans*** **protein name**	**Peptide**	***P. gingivalis*** **strain**	**Observed m/z ratio (charge)**	**Calculated mass [Da]**	**Score**	
						**+ Ox**	**- Ox**
P0CY35 (EF1A1_CANAL)*	Elongation factor 1-alpha 1, *Candida albicans* (strain SC5314 / ATCC MYA-2876)	^{254}IGGIGTVPVG**R**^{264}	—	513.3000 (+2)	1024.5854	59	73
			W83	513.8850 (+2)	1025.7554	40	
			Δ*ppad*	513.3790 (+2)	1024.7434	61	
P30575 (ENO1_CANAL)	Enolase 1, *Candida albicans* (strain SC5314 / ATCC MYA-2876)	^{319}IQIVGDDLTVTNPT**R**^{333}	—	548.0200 (+3)<br>821.4960 (+2)	1640.9774		115
			W83	821.9600 (+2)	1641.9054	26	46
			Δ*ppad*	821.4660 (+2)	1640.9174		108
Q92211 (G3P_CANAW)*	Glyceraldehyde-3-phosphate dehydrogenase, *Candida albicans* (strain WO-1)	^{227}LTGMSL**R**^{233}	—	389.2000 (+2)	776.3854	46	
			W83	389.7600 (+2)	777.5054	37	
			Δ*ppad*	389.2930 (+2)	776.5714	30	
		^{234}VPTTDVSVVDLTV**R**^{247}	—	750.9000 (+2)	1499.7854	152	95
			W83	751.5040 (+2)	1500.9934	62	51

			Δ*ppad*	750.9760 (+2)<br>501.0030 (+3)	1499.9374	94	88
		^{309}LISWYDNEYGYST**R**^{322}	—	883.9000 (+2)	1765.7854	101	
			W83	884.5000 (+2)	1766.9854	30	
			Δ*ppad*	883.9390 (+2)	1765.8634	68	
P41797 (HSP71_CANAL)*	Heat shock protein SSA1, *Candida albicans* (strain SC5314 / ATCC MYA-2876)	^{158}DAGTIAGLNVL**R**^{169}	—	600.4000 (+2)	1198.7854	79	55
			W83	600.9170 (+2)	1199.8194	59	
			Δ*ppad*	600.4170 (+2)	1198.8194	57	53
P46587 (HSP72_CANAL)*	Heat shock protein SSA2, *Candida albicans* (strain SC5314 / ATCC MYA-2876)	^{158}DAGTIAGLNVM**R**^{169}	—	609.4150 (+2)	1216.8154	84	
			W83	609.8810 (+2)	1217.7474	47	
			Δ*ppad*	609.3860 (+2)	1216.7574	60	
Q59XX2 (MP65_CANAL)	Cell surface mannoprotein MP65, *Candida albicans* (strain SC5314 / ATCC MYA-2876)	^{142}SESQIASEIAQLSGFDVI**R**^{160}	—	684.0000 (+3)<br>1025.5000 (+2)	2048.9854	152	
			W83	684.4070 (+3)<br>1026.1000 (+2)	2050.1854	239	69
			Δ*ppad*	684.1000 (+3)<br>1025.5000 (+2)	2048.9854	341	48
		^{311}NVLITETGWPS**R**^{322}	—	686.9270 (+2)	1371.8394	41	
			W83	687.4080 (+2)	1372.8014		35
			Δ*ppad*	686.9210 (+2)	1371.8274	66	
P28877 (PMA1_CANAX)*	Plasma membrane ATPase 1, *Candida albicans*	^{876}SLEDFLVSMQ**R**^{886}	—	662.9000 (+2)	1323.7854	78	
			W83	663.4000 (+2)	1324.7854	37	
			Δ*ppad*	662.9530 (+2)	1323.8914	90	
