# Supplementary material for: Peptidylarginine Deiminase of Porphyromonas gingivalis Modulates the Interactions between Candida albicans Biofilm and Human Plasminogen and High-Molecular-Mass Kininogen
Source: Int J Mol Sci. 2020 Apr 3;21(7):2495. doi: 10.3390/ijms21072495 (PMC7177930; doi:10.3390/ijms21072495)
Supplement: Supplementary file 1 [file ijms-21-02495-s001.zip › Supplementary Files revision/Supplementary File 3.pdf]

Table S3. ***C. albicans*** **proteins citrullinated after incubation with** ***P. gingivalis*** **PPAD.**

5x10^8 of *C. albicans* cells grown in RPMI 1640 medium for 18 hours were incubated with 0.1 µM *P. gingivalis* PPAD for 2 hours and then subjected to trypsin digestion to release peptides from fungal surface-localized proteins. The obtained peptides were then subjected to LC-MS/MS identification. The results from three independent experiments are presented (the asterisk denotes proteins identified as citrullinated for the first time in this study).

**Accession number**	***C. albicans*** **protein name**	**Peptide**	**PPAD**	**Observed m/z ratio (charge)**	**Calculated mass [Da]**	**Ion score**
P43067 (ADH1_CANAX)	Alcohol dehydrogenase 1, *Candida albicans*	^{303}DTAEAIDFFS**R**^{313}	-	636.3510 (+2)	1270.6874	61
			+	636.8780 (+2)	1271.7414	73
Q59L12 (ALS3_CANAL)*	Agglutinin-like protein 3, *Candida albicans* (strain SC5314 / ATCC MYA-2876)	^{167}ISINVDFE**R**^{175}	-	546.9000 (+2)	1091.7854	68
			+	547.3150 (+2)	1092.6154	67
		^{182}GYLTDS**R**^{188}	-	406.3000 (+2)	810.5854	51
			+	406.7030 (+2)	811.3914	46
Q5AIR7 (ENG1_CANAL)	Endo-1,3(4)-beta-glucanase 1, *Candida albicans* (strain SC5314 / ATCC MYA-2876)	^{431}AIDTNAPPTVFA**R**^{443}	-	686.9110 (+2)	1371.8074	71
			+	687.4050 (+2)	1372.7954	56
P30575 (ENO1_CANAL)	Enolase 1, *Candida albicans* (strain SC5314 / ATCC MYA-2876)	^{315}VGDKIQIVGDDLTVTNPT**R**^{333}	-	681.0940 (+3)	2040.2602	29
			+	681.6000 (+3)<br>1021.6000 (+2)	2041.3162	25
		^{319}IQIVGDDLTVTNPT**R**^{333}	-	821.4650 (+2)	1640.9154	75
			+	822.0400 (+2)	1642.0654	72
Q92211 (G3P_CANAW)*		^{234}VPTTDVSVVDLTV**R**^{247}	-	501.0070 (+3)<br>751.0130 (+2)	1499.9992	121

	Glyceraldehyde-3-phosphate dehydrogenase, *Candida albicans* (strain WO-1)		+	501.3580 (+3)<br>751.5150 (+2)	1500.9934	53
		[309]LISWYDNEYGYST**R**[322]	-	884.0010 (+2)	1765.9934	91
			+	884.5260 (+2)	1767.0734	64
Q59XX2 (MP65_CANAL)	Cell surface mannoprotein MP65, *Candida albicans* (strain SC5314 / ATCC MYA-2876)	[142]SESQIASEIAQLSGFDVI**R**[160]	-	684.1000 (+3)<br>1025.600 (+2)	2049.1854	117
			+	684.6000 (+3)<br>1026.1650 (+2)	2050.7782	116
Q5AJY5 (PGA4_CANAL)*	1,3-beta-glucanosyltransferase PGA4, *Candida albicans* (strain SC5314 / ATCC MYA-2876)	[69]YFQELGINTI**R**[79]	-	677.4250 (+2)	1352.8354	81
			+	678.0000 (+2)	1353.9854	44
P43076 (PHR1_CANAL)*	pH-responsive protein 1, *Candida albicans* (strain SC5314 / ATCC MYA-2876)	[201]QIPVGYSSNDDEEI**R**[215]	-	861.4820 (+2)	1720.949	58
			+	861.9930 (+2)	1721.9714	80
