# Supplementary material for: Peptidylarginine Deiminase of Porphyromonas gingivalis Modulates the Interactions between Candida albicans Biofilm and Human Plasminogen and High-Molecular-Mass Kininogen
Source: Int J Mol Sci. 2020 Apr 3;21(7):2495. doi: 10.3390/ijms21072495 (PMC7177930; doi:10.3390/ijms21072495)
Supplement: Supplementary file 1 [file ijms-21-02495-s001.zip › Supplementary Files revision/Supplementary File 4.pdf]

Table S4. Peptides identified for HPG that had been citrullinated by bacterial PPAD in the presence of gingipains.

150 nM human plasminogen was incubated with 50 nM PPAD and 1.5 nM HRgpA and Kgp for 18 hours at 37°C, and the peptides obtained after trypsin digestion were analyzed by LC-MS/MS. The theoretical mass in each case was calculated using PeptideMass software on the ExPASy server [89].

Peptide	Observed m/z ratio (charge)	Calculated mass [Da]	Theoretical mass [Da]	Ion score
^{81}KSSIII**R**^{87}	409.3000 (+2)	816.5854	815.52	29
^{82}SSIII**R**^{87}	345.3000 (+2)	688.5854	687.43	28
^{82}SSIIIRM**R**^{89}	488.8000 (+2)	975.5854	974.57	52
^{513}HSIFTPETNP**R**^{523}	650.4000 (+2)	1298.7854	1297.64	54
^{657}LFLEPT**R**^{663}	438.8000 (+2)	875.5854	874.49	39
^{732}YEFLNG**R**^{738}	450.3000 (+2)	898.5854	897.43	26
^{799}FVTWIEGVM**R**^{808}	619.9000 (+2)	1237.7854	1236.63	72
