# Supplementary material for: Peptidylarginine Deiminase of Porphyromonas gingivalis Modulates the Interactions between Candida albicans Biofilm and Human Plasminogen and High-Molecular-Mass Kininogen
Source: Int J Mol Sci. 2020 Apr 3;21(7):2495. doi: 10.3390/ijms21072495 (PMC7177930; doi:10.3390/ijms21072495)
Supplement: Supplementary file 1 [file ijms-21-02495-s001.zip › Supplementary Files revision/Supplementary File 5.pdf]

Table S5. Peptides identified for HK that had been citrullinated by bacterial PPAD in the presence of gingipains.

150 nM HK was incubated with 50 nM PPAD and 1.5 nM HRgpA and Kgp for 18 hours at 37°C. The peptides subsequently obtained after trypsin digestion were analyzed by LC-MS/MS. The theoretical mass in each case was calculated using PeptideMass software on the ExPASy server [89].

**Peptide**	**Observed m/z ratio (charge)**	**Calculated mass [Da]**	**Theoretical mass [Da]**	**Ion score**
^{43}KYNSQNQSNNQFVLY**R**^{58}	1002.6000 (+2)	2003.1854	2001.97	18
^{44}YNSQNQSNNQFVLY**R**^{58}	626.0000 (+3)	1874.9782	1873.87	48
^{188}QVVAGLNF**R**^{196}	502.9000 (+2)	1003.7854	1002.56	62
^{316}KYFIDFVA**R**^{324}	580.4000 (+2)	1158.7854	1157.62	68
^{317}YFIDFVA**R**^{324}	516.3000 (+2)	1030.5854	1029.53	52
^{381}RPPGFSPF**R**^{389}	531.3000 (+2)	1060.5854	1059.56	43
